# Detection of human herpesvirus 7 in conjunctival samples collected from individuals recovering from conjunctivitis

**DOI:** 10.3389/fopht.2025.1641991

**Published:** 2025-08-19

**Authors:** Andrew T.E. Hartwick, Christina E. Morettin, Jennifer S. Harthan, Meredith Whiteside, Ellen Shorter, Spencer D. Johnson, Mary K. Migneco, Christian K. Olson, Julia B. Huecker, Tammy Than, Mae O. Gordon

**Affiliations:** ^1^ College of Optometry, Ohio State University, Columbus, OH, United States; ^2^ Illinois College of Optometry, Chicago, IL, United States; ^3^ School of Optometry, University of California Berkeley, Berkeley, CA, United States; ^4^ Illinois Eye and Ear Infirmary, University of Illinois at Chicago, Chicago, IL, United States; ^5^ College of Optometric Medicine, Rocky Mountain University, Provo, UT, United States; ^6^ School of Medicine, Washington University, St. Louis, MO, United States; ^7^ Fort Sam Houston, San Antonio, TX, United States; ^8^ Carl Vinson Veteran Affairs Medical Center, Dublin, GA, United States

**Keywords:** conjunctivitis, herpes, HHV-7, virus, adenoviral

## Abstract

**Purpose:**

Although it is often reported that adenovirus is the most common etiology for infectious conjunctivitis, a recent multi-center clinical study found that adenovirus was confirmed by polymerase chain reaction in only 16% of cases presenting with acute conjunctivitis. Here, we investigated the hypothesis that a member of Herpesviridae could be the underlying etiology in some non-adenoviral cases of conjunctivitis.

**Methods:**

Molecular assays for Herpes Simplex 1 and 2 (HSV-1, HSV-2) and Human Herpesvirus 6A, 6B and 7 (HHV-6A, HHV-6B, HHV-7) were performed on conjunctival samples collected from 18 individuals with acute conjunctivitis and during their recovery in follow-up visits that spanned up to 3 weeks. All samples, obtained from individuals enrolled in a clinical trial evaluating a conjunctivitis treatment, were from eyes that had previously tested negative for adenovirus using polymerase chain reaction (PCR) techniques.

**Results:**

In total, 160 PCR assays were performed on 40 conjunctival samples. Four of these samples, obtained from four different individuals, tested positive for HHV-7. None of the samples tested positive for HSV-1, HSV-2, HHV-6A or HHV-6B.

**Conclusion:**

This data provides further evidence that Human Herpesvirus 7 can be present in the eye, as HHV-7 was detected in a subset of conjunctival samples obtained from individuals recovering from non-adenoviral conjunctivitis. Clinicians should consider non-adenoviral etiologies when managing conjunctivitis that presents as classic ‘pink eye’.

## Introduction

Infectious conjunctivitis is an extremely prevalent ocular condition (1) that causes significant discomfort to affected individuals during its acute phase (2). It is often reported that adenovirus is the most common etiology for infectious conjunctivitis, with this condition being colloquially referred to as ‘pink eye’ (3). However, in a recent study in which quantitative polymerase chain reaction (qPCR) was performed on conjunctival samples obtained from 186 screened individuals with suspected pink eye, it was found that only 30 out of 186 (16.1%) had molecularly-confirmed adenovirus present (4). Furthermore, adenovirus was detected by qPCR in only 50% of the samples that had tested positive for adenovirus on a point-of-care immunoassay (4), raising the possibility that the antigen-based assay was being activated by a different virus present in the conjunctival samples.

To explore this premise, samples collected from seven participants with acute conjunctivitis were sent for processing by an independent laboratory that utilizes metagenomic shotgun sequencing approach (5) to identify potential viral populations. These individuals had been enrolled in a randomized clinical trial that investigated the efficacy of povidone-iodine for adenoviral conjunctivitis (2), and the samples had all tested positive for adenovirus on a point-of-care immunoassay but negative for that virus through qPCR analysis. A DNA fragment consistent with the Herpes virus family (*Herpesviridae*), and specifically the genus *Roseolovirus*, was detected in one of these samples using this metagenomic DNA sequencing approach.

Although these preliminary results were suggestive of a herpetic etiology in a case of suspected pink eye, metagenomic shotgun sequencing results can be affected by erroneous reads that could lead to misidentification of parent organisms (6). To test the hypothesis that a member of *Herpesviridae* could be the underlying etiology in these eyes, PCR assays for Herpes simplex 1 and 2 (HSV-1, HSV-2; commonly associated with ocular infections) and human herpesvirus 6A, 6B and 7 (HHV-6A, HHV-6B, HHV-7; members of the genus *Roseolovirus*) were performed on conjunctival samples collected from individuals presenting with and recovering from acute conjunctivitis. These samples, previously obtained from individuals enrolled in a clinical trial assessing conjunctivitis treatment (2), had already tested negative for adenovirus using PCR techniques. Therefore, this study aimed to investigate potential non-adenoviral etiologies for conjunctivitis that presents with symptoms similar to that for classic ‘pink eye’.

## Methods

This study represents analyses performed on conjunctival samples collected during a larger parent clinical trial that assessed the efficacy of povidone-iodine (PVP-I) for adenoviral conjunctivitis. The primary outcomes of that trial have been published (2, 4, 7). Institutional review board (IRB) approval was obtained from all participating clinics and the study complied with the ethical principles of the Declaration of Helsinki and Good Clinical Practices. Adults (age ≥18 years) presenting with red eye(s) and symptom duration of 4 days or less were invited for eligibility screening. Exclusion criteria included recent ocular surgery, skin vesicles, corneal dendrites, conjunctival membrane or pseudomembrane, corneal infiltrates, corneal ulceration, corneal abrasion, ocular foreign body, or anterior chamber inflammation.

Participants completed a standardized eye examination that included slit lamp examination of the anterior segment by a clinician who graded clinical signs such as bulbar conjunctival redness, serous discharge, and the palpebral conjunctival follicular response. Point-of-care immunoassay testing for adenovirus (AdenoPlus, now named QuickVue Adenoviral Conjunctivitis Test; Quidel Corporation, San Diego, CA, USA) was performed and eligible participants with positive AdenoPlus test results were enrolled in the treatment trial, in which they randomly were assigned to a lavage treatment of either povidone-iodine or saline (see ref. 2 for details). Inferior palpebral conjunctival swab samples, using flocked sterile swab applicators (Becton Dickinson, Sparks MD), were taken at the presenting visit (Day 0) and up to 5 subsequent follow-up visits (Day 1-2, 4-5, 7, 14 and 21). If both eyes showed signs of conjunctivitis, the first eye with reported symptoms was the one that the conjunctival samples were obtained from. Swabs were placed in Universal Viral Transport medium (Becton, Dickinson and Company) and frozen at −80°C within 4 hours of collection. The samples were shipped on dry ice in batches to the Coordinating Center (Washington University), where they were stored at −80°C for several months prior to molecular analyses that were performed after the parent clinical trial had concluded.

For this study, 40 conjunctival samples, which were obtained from the eyes of 18 enrolled participants that entered the clinical trial with acute conjunctivitis, were shipped to a private laboratory (Eurofins Viracor, Lenexa, KS) for PCR detection of DNA consistent with either HSV-1, HSV-2, HHV-6A, HHV-6B or HHV-7. Consistent with the eligibility criteria listed above, all 18 participants had tested positive on the point-of-care adenovirus immunoassay (AdenoPlus) at the initial visit. However, no adenovirus had been detected in any of these samples following subsequent qPCR testing, and so the underlying etiology for the conjunctivitis was unknown for these participants. The samples from these 18 participants were chosen for this study because they were the remaining samples from adenoviral-negative eyes that had been collected in a multi-center clinical trial (2) on conjunctivitis. The mean age for the 18 participants was 33.4 years (± 13.4 SD; range 18 to 59), and this participant cohort came from four different USA-based study sites. Ten of the 18 participants were female, and the self-reported racial breakdown was: 11 White, 4 African American and 3 individuals who reported ‘Other’.

Up to 4 samples per participant were assayed, which included those obtained at the initial (Day 0) and three follow-up (Day 1-2, Day 4-5, Day 21) visits. All 4 samples were not available for every participant due to either missed follow-up visits, or lack of sample due to its use in prior separate molecular analyses. Many of the Day 0 conjunctival samples collected during the randomized clinical trial had been completely used for other analyses separate from the present study, and so initial Day 0 samples were only available for 7 of the 18 individuals.

The individual primers and probes for HSV-1 and HSV-2 are reported by Eurofins Viracor to be unique and specific, with no cross reactivity between the HSV-1 and HSV-2 primers, nor against adenoviruses, BK virus (BKV), cytomegalovirus (CMV), Epstein-Barr virus (EBV), HHV-6A, HHV-6B, HHV-7, HHV-8, John Cunningham virus (JCV), parvovirus B19, simian virus-40 (SV-40), and varicella zoster virus (VZV). The HHV-6 assay is reported to detect both Type A and Type B variants, and it and the HHV-7 assay are reported to show no cross reactivity between each other or against adenoviruses, BKV, CMV, EBV, HSV-1, HSV-2, HHV-8, JCV, parvovirus B19, SV-40, and VZV.

## Results

In total, 160 PCR assays were performed for this study as each of the 40 samples was tested for HSV-1, HSV-2, HHV-6A/6B and HHV-7, respectively. The 40 assays for each of HSV-1, HSV-2 and HHV-6A/6B were all negative. Out of the 40 HHV-7 assays, 36 were negative and 4 were positive. The positive samples were obtained from 4 different participants, and there was no pattern in terms of the visit that the positive sample was obtained (**Table 1**). None of the 4 participants had multiple positive samples across different visits. The mean age of the four HHV-7-positive individuals was 38.0 years (range 18 to 58; mean age of remaining 14 participants was 32.1, range 19 to 59). As the participants were enrolled in a randomized treatment trial, 3 of the 4 participants with HHV-7 positive conjunctival samples received povidone-iodine (PVP-I) treatment at the initial visit (Day 0), while one participant was in the control arm and received saline treatment instead (**Table 1**).

**Table 1:** Results from PCR assays for HHV-7 performed on conjunctival samples obtained from the 4 of 18 participants that had one positive result for HHV-7.

HHV-7 PCR Assay					
Participant	Treatment at Day 0	Day 0	Day 1-2	Day 4-5	Day 21
1	PVP-I	**Positive**	Negative	Negative	N/A
2	Saline	N/A	**Positive**	Negative	Negative
3	PVP-I	N/A	Negative	**Positive**	Negative
4	PVP-I	N/A	Negative	Negative	**Positive**

The Day 0 samples were not available for participants 2, 3, 4 due to use in prior molecular analyses. The Day 21 sample was not available for participant 1 due to a missed follow-up visit. Participants 1, 3, 4 received PVP-I treatment, while participant 2 received saline, at the initial visit.

N/A, sample unavailable to be tested. Bolded results represent samples that tested positive for HHV-7.

At the initial visit, the examining clinicians were asked whether they thought the conjunctivitis in each participant had an adenoviral etiology (response choices: ‘definitely yes’, ‘probably yes’, ‘probably not’, ‘definitely not’). The clinicians answered ‘probably yes’ for participants 1 and 3, and ‘definitely yes’ for participants 2 and 4. This survey indicates that the initial clinical presentation of the four cases was consistent with that typically associated with ‘pink eye’, based on the clinical judgement of the examiners.

The severity of three clinical signs (bulbar redness, serous discharge and follicular response) on the initial visit (Day 0), Day 1-2, Day 4-5, and Day 21 visits (**Table 2**) was examined for each of the four HHV-7-positive cases. These 3 signs were graded by clinicians, who were masked to whether the participants were treated with PVP-I or saline, on a 4-point scale. The clinicians graded seven clinical signs on this scale during their slit lamp examination in the clinical trial (2), but these three received the highest grades during the initial visits and were therefore chosen here to examine potential differences in the recovery time-course. For comparison, the mean grades for participants in the clinical trial with qPCR-confirmed adenoviral conjunctivitis (2) are included in the bottom rows (**Table 2**). The data listed is that for the group of participants that were treated with PVP-I on Day 0, after the clinical signs had been graded for that visit, because 3 of the 4 participants testing positive for HHV-7 were similarly treated with PVP-I (the saline-treated group with adenoviral conjunctivitis had significantly higher mean grades for each of these 3 clinical signs at the Day 4–5 visit; see ref. 2). The grades for the four HHV-7-positive cases tended to be lower than the mean grades for the group with confirmed adenoviral conjunctivitis, particularly at the first two follow-up visits.

**Table 2:** Gradings of bulbar redness, serous discharge and follicular response by the examining clinician for the 4 participants with a positive HHV-7 result at the initial (Day 0) and follow-up (Day 1-2, Day 4-5, Day 21) visits.

Day 0 Visit				Day 1–2 Visit			
Participant	Bulbar Redness	Serous Discharge	Follicular Response	Participant	Bulbar Redness	Serous Discharge	Follicular Response
1	3	3	2	1	3	2	2
2	4	3	4	2	4	3	4
3	4	3	2	3	3	2	2
4	5	5	4	4	3	3	3
PVP-I Treated Ad-Cs (n=16)	4.0 ± 0.6	3.4 ± 1.1	3.4 ± 1.0	PVP-I Treated Ad-Cs (n=13)	4.0 ± 0.8	3.3 ± 1.0	3.1 ± 1.1

Day 4–5 Visit				Day 21 Visit			
Participant	Bulbar Redness	Serous Discharge	Follicular Response	Participant	Bulbar Redness	Serous Discharge	Follicular Response
1	2	1	1	1	N/A	N/A	N/A
2	1	1	2	2	1	1	1
3	1	1	2	3	1	1	1
4	1	1	2	4	1	1	1
PVP-I Treated Ad-Cs (n=8)	2.7 ± 0.8	2.0 ± 1.0	2.7 ± 1.0	PVP-I Treated Ad-Cs (n=10)	1.7 ± 0.9	1.0 ± 0.0	1.2 ± 0.3

Grading done on a 4-point scale (1 to 4). The mean grades (from ref. 2) for the PVP-I-treated group with PCR-confirmed adenoviral conjunctivitis are shown for comparison.

## Discussion

HSV-1, HSV-2 and HHV-6 have been previously reported as causative agents for conjunctivitis (8, 9). In the conjunctival samples collected (at initial and follow-up visits occurring over 3 weeks) from 18 individuals recovering from suspected infectious conjunctivitis, the PCR assays for HSV-1, HSV-2, HHV-6A and HHV-6B were all negative. Samples from these same individuals had previously tested negative for adenovirus by PCR in analyses performed for the parent clinical trial that these individuals had been enrolled in (2). For this parent study, an inclusion criterion was that all enrolled participants had to test positive for adenovirus on the AdenoPlus point-of-care immunoassay, and so the subsequent negative PCR testing for adenovirus indicated that the immunoassay tests for these 18 individuals reflected a false positive result (4). Thus, the results of the present study do not support the hypothesis that the previous false-positive results on the point-of-care immunoassays could be attributed to these specific herpetic viruses.

However, the PCR analysis did result in the detection of HHV-7 in 4 samples, collected from four different individuals. HHV-7 is typically thought to be acquired in childhood and can cause roseola, febrile seizures and encephalitis (10). To our knowledge, this virus has not been associated with infectious conjunctivitis. As the positive results were obtained at different visit dates, including one that occurred 3 weeks after the initial visit and after complete resolution of clinical signs and symptoms, the data did not conclusively identify HHV-7 as the likely causative agent for the conjunctivitis.

There are a couple of possible interpretations for these results. One is that HHV-7 was the underlying etiology for the conjunctivitis in these individuals and present in their eyes at most of the visits, but the viral load may have been relatively small and near the threshold of detection for PCR assay. The laboratory reports that the assay is sensitive enough to detect 51 DNA copies/ml for cerebrospinal fluid samples, but the threshold has not been determined for ocular samples (personal communication with Eurofins-Viracor). Therefore, whether the results were positive or negative may have been affected by inherent variability in the swab collection technique, with positive results being obtained when more conjunctival cells were collected.

A second possibility is that HHV-7 was present in the four individuals prior to the conjunctivitis, and so its detection was incidental to the concurrent pathology. As the primary HHV-7 infection typically occurs in childhood (10), and all the participants in this study were adults, reactivation of latent HHV-7 may have been triggered during the acute conjunctivitis that was caused by a non-herpetic (and non-adenoviral) agent. The presence of the HHV-7 DNA virome is commonly found in human blood samples (11), likely due to a prior childhood infection, and the virus is commonly reactivated in immunocompromised patients such as transplant recipients (12). In this scenario, it is possible that the inflammation that occurred in these individuals during the acute conjunctivitis triggered a rise in the virus present in ocular tissues, as HHV-7 has been shown to remain latently present in lymphocytes in previously infected individuals (13). It is our belief that the most parsimonious explanation is likely this latter interpretation. The decision to assay samples collected at multiple visits in this study was based on the premise that the causative agent would be detected at the early visits (Day 0, Day 1–2 and possibly Day 4-5) and then absent at the Day 21 visit when the clinical signs and symptoms had since resolved. The seemingly random pattern of visits in which the positive HHV-7 results were observed in this study provides a relatively strong argument against HHV-7 being the underlying etiology in these cases of conjunctivitis.

The clinical presentation of these four cases at the initial visit was not overly different from other participants with acute conjunctivitis who were enrolled in the larger parent clinical treatment trial. All four cases from whom the positive HHV-7 samples were obtained were judged to ‘probably’ or ‘definitely’ be adenoviral in nature by the examining clinician at the initial visit. As illustrated in **Table 2**, the major clinical signs associated with pink eye (bulbar redness, serous discharge, follicular response) were all present at the initial visit and rated at a similar severity to those for the cases with PCR-confirmed adenoviral conjunctivitis. The severity grades for these signs given by clinical examiners at the follow-up visits suggest that these four cases perhaps may have exhibited quicker resolution than that experienced by participants with true adenoviral conjunctivitis. This is consistent with the findings from the parent clinical trial in which these individuals were enrolled: the natural history of true adenoviral conjunctivitis typically involves signs and symptoms that persist for over a week, whereas non-adenoviral conjunctivitis (likely heterogeneous etiologies) often resolves more quickly (14).

A major finding of this work is that HHV-7 is present in conjunctival samples obtained from a subset of individuals who present with acute conjunctivitis or for whom conjunctivitis has recently resolved. In a previously published case report, HHV-7 was detected in aqueous humor obtained from a patient with corneal endotheliitis (15). HHV-7 has also been detected in a minority of conjunctival samples collected from patients with lymphoproliferative disorders such as cancerous lymphoma of the ocular adnexa (13). Our data therefore bolsters the evidence that HHV-7 can be detected in anterior ocular tissue/fluid, particularly in certain individuals with anterior ocular inflammation. There is a report of HHV-7 DNA being identified in the vitreous fluid of a single individual with infectious uveitis due to toxoplasmosis (16), but this does not appear to be a common finding as it was not present in vitreal samples collected from over 100 patients with retinitis or posterior uveitis in a separate study (17).

In summary, this data provides evidence that HHV-7 can be detected in the conjunctiva in a subset of individuals presenting or recovering from non-adenoviral conjunctivitis. The causative microbial agent underlying these cases of conjunctivitis, which produce positive test results on adenoviral immunoassays despite being negative for this virus upon PCR testing, remains unknown but our results here do not support a herpetic etiology (HSV-1, HSV-2, HHV-6A/6B, HHV-7) for these cases. The overall prevalence of HHV-7 in the conjunctiva in healthy individuals also remains unknown, but these results raise the possibility that its detection may be influenced by recent infection by another pathogen.

## Data Availability

The raw data supporting the conclusions of this article will be made available by the authors, without undue reservation.
